# Sentiment Contagion Based on the Modified SOSa-SPSa Model

**DOI:** 10.1155/2016/9682538

**Published:** 2016-09-22

**Authors:** Zhijie Song, Rui Shi, Jie Jia, Jian Wang

**Affiliations:** School of Economic and Management, Yanshan University, Qinhuangdao, China

## Abstract

Sentiment contagion is similar to an infectious disease that spreads in a crowd. In this study, we extend the proposed SOSa-SPSa model (susceptible-optimistic-susceptible and susceptible-pessimistic-susceptible) by considering the interaction between optimists and pessimists. Simulation results show that our model is reasonable and can better explain the entire contagion process by considering three groups of people. The recovery speed of pessimists has an obvious regulative effect on the number of pessimists and the possibility of optimists coming in contact with pessimists to be infected as pessimism plays a greater role than that of reverting to susceptibility. The number of pessimists is positively related to the possibility that optimists come in contact with pessimists to become pessimistic but is negatively related to the possibility of the other way around. When the speed of spontaneous generation is slow, the number of pessimists sharply increases. However, the increase is not so apparent when the speed of spontaneous generation reaches a certain number.

## 1. Introduction

Sentiment contagion is an interaction process in which individuals capture and imitate the emotion of others unconsciously. Through this period, individuals realize a synchronous state with others and attain aggregation. Sentiment contagion is the product and foundation of human society. The features of the entire contagion process are universal, unconscious, and convergent [1]. Previous studies on sentiment contagion mainly focused on individual level, that is, the process of being influenced by others. Through the entire process, we discuss the mechanism and procedure in which individuals are influenced by others, as well as the relationship between individual variation and sentiment contagion intensity [2]. Researchers have recently been concentrating on the interaction among community members. They have been turning their attention to the effect that sentiment contagion has on group behaviors [3]. Group behaviors are the indication of certain information and irrational behaviors caused by crowd sentiment contagion [4]. Human group behaviors occur in a large social network. People come in contact with one another, and this contact is helpful in changing behaviors in fundamental ways. These behaviors divert from public health, economics, and psychology to sociology. The behaviors of these fields are mostly driven by the inner desire of the human body and affected by emotion.

The following studies examined the behaviors of the abovementioned fields. Emergencies have occurred many times without any sign and caused severe damage throughout the entire country. In a normal condition, these unexpected disasters trigger public disorder and easily cause public panic or even social unrest [5–7]. Huang (2014) uses an ERP (Event Related Potential) experiment to study the public sentiment of panic under unconventional emergencies, and the obtained physiological indexes can be the measurement of sentiment. These indexes are of great help for managers to supervise and establish a monitoring system [8]. The contagion and spread of investor sentiment are currently being extensively discussed. The sentiment reaction among investors may induce the instability of the stock market, and the spread of the investor sentiment may cause herd behaviors, information block, price bubble, and unusual fluctuation in stock price. All of these phenomena have greatly influenced the effectiveness of the financial market and normal operation. Zhang et al. (2007) identifies that the fluctuation in investor sentiment asymmetrically affects the fluctuation in stock prices. The change in stock prices actually depends on the positive or negative changes in investor sentiment [9]. Cui (2013) discovers remarkable correlativity between investor sentiment contagion and market rationality [10].

Research on the mechanism of sentiment contagion is mostly conducted through models originating from the classical model of SIR or SIS [11–14]. These two models are designed to study the infection and spread of a disease. Fu et al. (2014) introduce the CA-SIRS model. They captured the dynamic process of susceptible-infected-recovered-susceptible and finds that the proportion of initial infected individuals insignificantly affects the final stable proportion of infected population in a given system and that the infection frequency increases with an increase in the average crowd density [15]. In the research of emotion contagion in a large social network, Zhao et al. (2014) discovered that the overall tendency of sentiment variation in a BA-scale free network is similar to that in a homogeneous network and that the assimilation and weight combination affects sentiment contagion [16]. Sentiment contagion is also one aspect of social contagion that contains spontaneous factors other than transmission. Experts introduce a novel form of the classical susceptible-infected-susceptible disease model, which includes the possibility for “spontaneous” infection. Thus, Hill et al. (2010) first extended the SIS model by adding a process in which uninfected individuals become infected subjects spontaneously at a constant rate *α*, regardless of whom the uninfected individuals come in contact with [17, 18]. The constitution of the crowd in sentiment contagion is complex. Consequently, barely classifying optimists and pessimists as infected people is not sufficient to understand the mechanism of sentiment contagion. Thus, Liu et al. (2014) divided the infected status into two states, namely, optimistic subjects (*O*) and pessimistic subjects (*P*), based on the SISa model. They developed the SISa model into the SOSa-SPSa model to study sentiment contagion [19].

Overall, the study of sentiment contagion is of great importance in the field of emergencies and the stock market, both of which need further study, especially in the deeper mechanism of contagion and the interactions of groups possessing different emotion states. However, the limitation of the SOSa-SPSa model is that it only assumes that optimists and pessimists can only transform into susceptible subjects but cannot interact with one another directly. In reality, especially in emergencies, the emotion of people is greatly influenced. Therefore, a certain possibility exists for pessimists to come in contact with optimists. Examining their interaction is essential. Based on the predecessor study, this study analyzes the sentiment contagion process based on the SOSa-SPSa model by considering the transformation mechanism between optimism and pessimism to further expose the deeper rules of sentiment contagion.

This paper is divided into four sections.  Section 1 contains the background and a brief introduction of the research status.  Section 2 presents our modified model.  Section 3 provides the equilibrium result of the modified model and the simulation results of different parameters. Finally, Section 4 concludes the study and describes its drawbacks and direction for further relevant study.

## 2. Modified SOSa-SPSa Model Considering the Interaction between Optimists and Pessimists

The two basic and the most classic epidemic models, SIS and SIR, can be used to describe the process and propagation rule of a disease. In the SIS model, the crowd can be divided into two states, namely, susceptible subjects (*S*) and infected subjects (*I*). The model depicts the interaction of the two states and does not confer immunity. The other model is SIR, in which the crowd is classified into three groups, namely, susceptible subjects (*S*), infected subjects (*I*), and removal (*R*). The difference between the two models is that the susceptible people in the SIS model come in contact with the infected people and become infected. After being treated, these recovering people return to being susceptible again. Otherwise, the recovering people in the SIR model become immune to further infection and enter the removal state. Sentiment can be regarded as an infectious disease. The characteristics of sentiment contagion are similar to those of an infectious disease.

**Figure 1:**
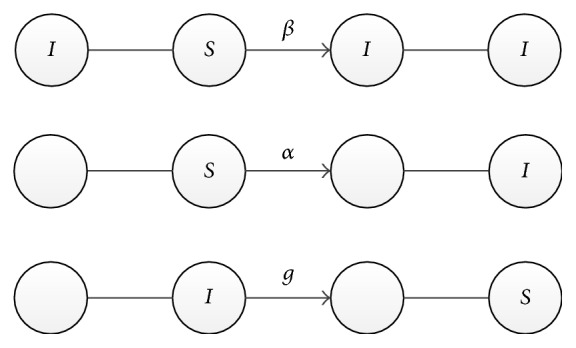
Rule of contagion of SISa.

The SISa model comprises three processes, where *S* and *I* represent the susceptible people and the infected people, respectively, *β* is the transmission speed, *g* is the recovery speed when infected people return to the state of suspected people, and *α* is the spontaneous infection rate. The diagrammatic representation is presented in Figure 1.

Based on the rules, all emotions are classified into one type. Mood is not classified according to mood valence. However, in a real situation, mood is also divided into positive and negative emotions. This classification is not a comprehensive description of the dynamic change process of emotional contagion.

The SOSa-SPSa model includes two conversion processes, namely, (i) susceptible-optimistic-susceptible (SOS) and (ii) susceptible-pessimistic-susceptible (SPS), both of which include a spontaneous process.

**Figure 2:**
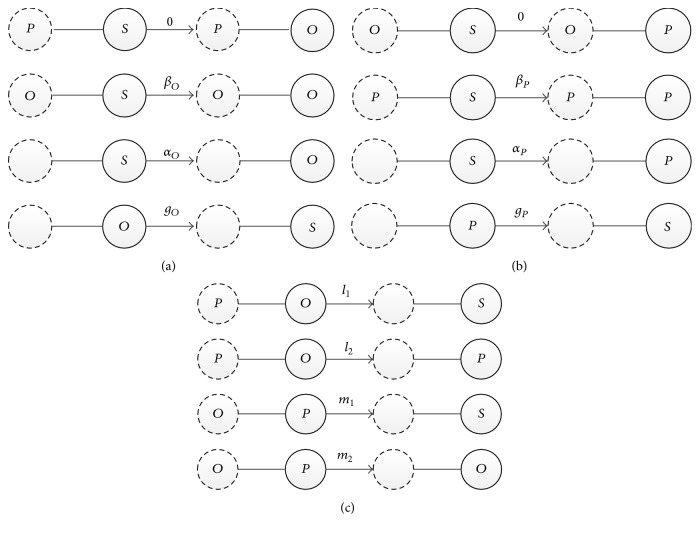
(a) Process of sentiment contagion between the optimistic and the susceptible subjects. (b) Process of sentiment contagion between the pessimistic and the susceptible subjects. (c) Process of sentiment contagion between the pessimistic and the optimistic subjects.

The refined SOSa-SPSa model in Figure 2(a) depicts the process of sentiment contagion between the optimistic subjects and the susceptible subjects as follows: (1) a small possibility exists for the susceptible subjects to become optimistic subjects when coming in contact with the pessimistic subjects; (2) when the optimistic subjects comes in contact with the susceptible subjects, the optimistic subjects remain optimistic, and the susceptible subjects become optimistic with a possibility of *β*_*O*_; (3) regardless of whom the susceptible subjects come in contact with, the susceptible subjects spontaneously become optimistic at a rate of *α*_*O*_; (4) regardless of whom the optimistic subjects come in contact with, the possibility of *g*_*O*_ that the optimistic subjects become susceptible exists.

Figure 2(b) presents the sentiment contagion process between the pessimistic subjects and the susceptible subjects. The four processes are as follows: (1) The susceptible subjects do not become pessimistic when coming in contact with the optimistic subjects. Therefore, the transition probability is 0. (2) Regarding the susceptible subjects confronting the pessimistic subjects, the state of the pessimistic subjects remains stable, while the susceptible subjects revert to being pessimistic at a rate of *β*_*P*_. (3) The susceptible subjects are spontaneously infected by the pessimistic subjects at a rate of *α*_*P*_ regardless of contacts. (4) Once infected as pessimistic subjects, the infected subjects recover at *g*_*P*_ speed regardless of contacts.

Figure 2(c) illustrates the interaction between the pessimistic subjects and the optimistic subjects. The situation can be generally divided into four categories. When the optimistic subjects come in contact with the pessimistic subjects, predicting who the pessimistic subjects will revert to is complicated. Nevertheless, two situations can be utilized to confirm which state the optimistic vwill turn to when coming in contact with the pessimistic subjects, namely, the susceptible subjects and the pessimistic subjects. The possibility of becoming the susceptible subjects is *l*_1_, and the possibility of becoming the pessimistic subjects is *l*_2_. The contagion process is also applicable to the pessimistic subjects to come in contact with the optimistic subjects. Regardless of who the optimistic subjects will revert to, the pessimistic subjects could become the susceptible subjects and the optimistic subjects via two possible ways. The possibility of reverting to the susceptible subjects is *m*_1_, and the possibility of being infected subjects as optimistic is *m*_2_.

**Figure 3:**
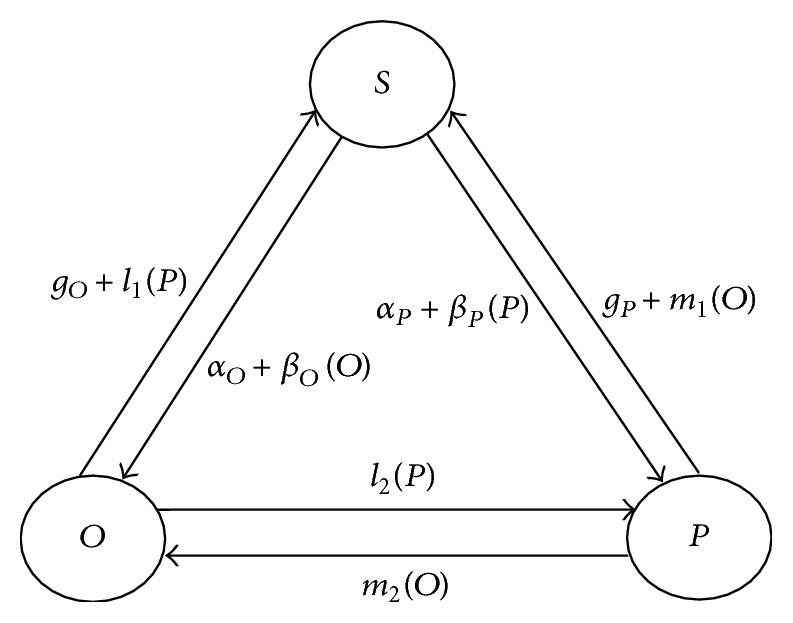
Basic framework of the sentiment contagion in the refined SOSa-SPSa model.

Figure 3 depicts the basic framework of the sentiment contagion among the pessimistic subjects, the optimistic subjects, and the susceptible subjects. The emotional contagion model in emergency explores the positive emotions, negative emotions, and emotional interaction among the susceptible subjects in the infection process. On the basis of the extended SISa model, we add positive and negative emotions to the crowd interaction relationship among groups to examine the mood-changing rule of the entire crowd when an individual comes in contact with another individual with an opposite emotion. Based on the extended SISa model, the SOSa-SPSa model, which is the refined model discussed in this paper, supplements the interaction between the optimistic subjects and the pessimistic subjects and the transition from the optimistic subjects or the pessimistic subjects to the susceptible subjects. Two transformation paths exist. One path is from susceptible subjects to transform into optimistic ones and then from optimistic subjects to transform to susceptible and pessimistic ones. The other path is from susceptible subjects to transform to pessimistic ones and then from pessimistic subjects to transform to susceptible and optimistic ones.

We add the parameters *l*_1_, *l*_2_, *m*_1_, and *m*_2_ to explore the influence of the interaction between the optimistic subjects and the pessimistic subjects on the mechanism of sentiment contagion and to distinguish which status the infected is prone to transfer when contacting with the opposite status. We hypothesize that the total number of the crowd subjects is *N*, among which the number of the pessimistic subjects is *P*, the number of the optimistic subjects is *O*, and the number of the susceptible subjects is *S*. Combining these numbers, we obtain the equation *P* + *O* + *S* = *N*. We also assume that the two processes of transition from the susceptible subjects to the pessimistic subjects and the optimistic subjects are independent from each other. The differential equations of the modified model are as follows:

(1) dSdt=gOO+gPP−αO+αPS−βOO+βPPS+l1+m1OP,dOdt=−gOO+αOS+βOOS−l1+l2−m2OP,dPdt=−gPP+αPS+βPPS−m1+m2−l2OP,O+P+S=N,l1+l2≤1;  m1+m2≤1.

To reach equilibrium, each equation shall be equal to zero; that is, d*S*/*dt* = 0, d*O*/*dt* = 0, and d*P*/*dt* = 0. The equilibrium point is then obtained. When *α*_*O*_ + *β*_*O*_, *O* ≠ 0 and *α*_*P*_ + *β*_*P*_, *P* ≠ 0, *S* = *g*_*P*_ *P* + *OP*(*m*_1_ + *m*_2_ − *l*_2_)/*α*_*P*_ + *β*_*P*_ *P* = *g*_*O*_ *O* + *OP*(*l*_1_ + *l*_2_ − *m*_2_)/*α*_*O*_ + *β*_*O*_ *O*. As a result, we generate the following equilibrium:

(2) $$\begin{array}{c} \frac{g_{P}P+OP\left(m_{1}+m_{2}-l_{2}\right)}{\alpha_{P}+\beta_{P}P}=\frac{g_{O}O+OP\left(l_{1}+l_{2}-m_{2}\right)}{\alpha_{O}+\beta_{O}O}. \end{array}$$

## 3. Model Simulation 

### 3.1. Simulation of the Sentiment Contagion Process

**Figure 4:**
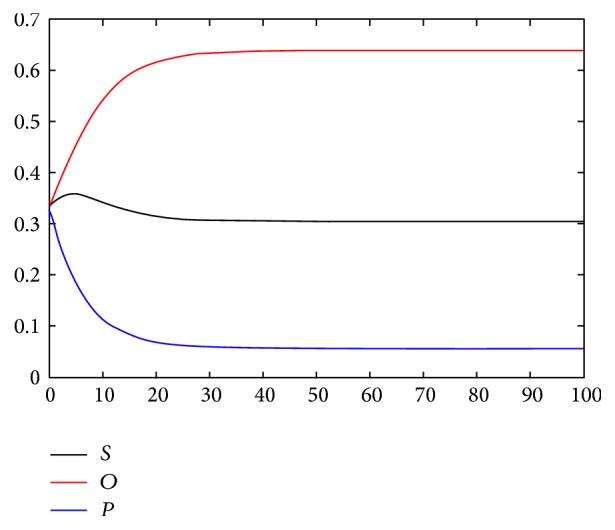
Changing process of the components of sentiment contagion.

In this section, we use MATLAB to simulate the differential equations to understand the mechanism of sentiment contagion and to conduct prediction and interventions. In the modified model, we set the parameters as those observed in the FHS data according to the results in the study of Hill et al. (2010); namely, *α*_*O*_ = 0.18, *β*_*O*_ = 0.02, *g*_*O*_ = 0.088, *α*_*P*_ = 0.04, *β*_*P*_ = 0.04, *g*_*P*_ = 0.13, *l*_2_ = 0.009, *m*_2_ = 0.07, *l*_1_ = 0.13, and *m*_1_ = 0.09. The initial values of the component in the crowd are *O* = 626, *P* = 626, and *S* = 628, and the total number of the crowd subjects is *N* = 1880. The simulation result shows that the number of the optimistic subjects increases, whereas the number of the pessimistic subjects decreases at the initial stage of sentiment contagion. The susceptible fluctuates in a small scope. An initial transient increase and a subsequent gradual decrease occur. The changing rate of the three components finally remains 0. The entire system remains stable.  Figure 4 presents that the final composition of the system is that the amount of the optimistic subjects takes up 63%, the pessimistic accounts for 7%, and the susceptible occupies almost 30%. The number of the entire system subjects is stable. The equilibrium state is finally reached, and the set of the parameters has no influence on the final equilibrium results. This finding matches that of Hill et al. (2010) and proves the rationality of the model.

### 3.2. Different Effects of Parameters on Equilibrium

**Figure 5:**
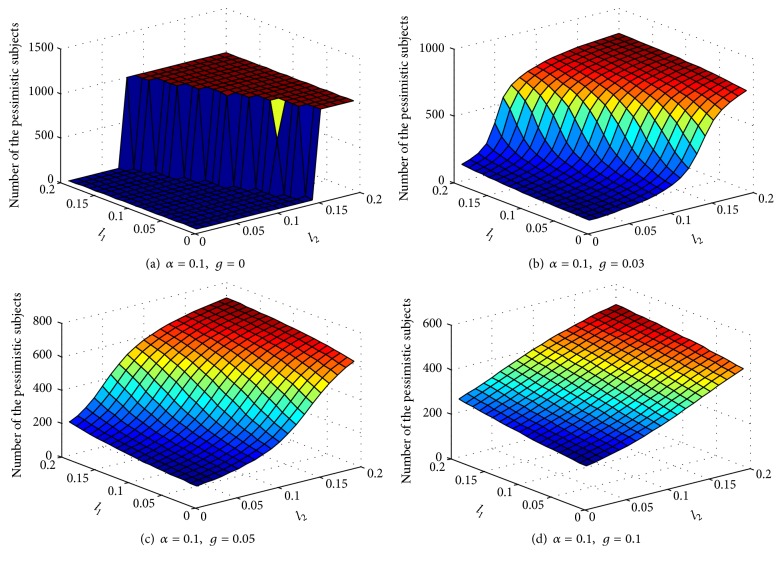
Effect of the recovery speed of the pessimistic on equilibrium.

Figure 5 depicts how the number of the pessimistic subjects in equilibrium varies with the changes in the recovery speed of the pessimistic. The number of the pessimistic subjects in the final equilibrium decreases as the recovery speed of the pessimistic increases. The contact of a pessimistic crowd with an optimistic crowd produces a great number of susceptible subjects, which then increases the proportion of the population infected with a negative emotional state. A fast recovery speed of the pessimistic leads to a small number of the final pessimistic subjects in equilibrium similar to our prediction. The angle of inclination becomes gentle with an increase in the recovery speed of the pessimistic subjects. Changes are not obvious, thus implying that the recovery speed of the pessimistic subjects has an obvious regulative effect. We aim to determine the effect of the pessimistic subjects on the optimistic subjects if a great possibility exists for the optimistic subjects to become pessimistic subjects when coming in contact with the pessimistic subjects. The comparison of *l*_1_ with *l*_2_ indicates that when the contagion speed to become pessimistic subjects *l*_2_ is faster than the contagion speed to become susceptible *l*_1_, the former holds a dominant position rapidly, and the number of the pessimistic subjects sharply increases to an equilibrium status.

The recovery speed of the optimistic is of great help to release the explosion of the number of the pessimistic subjects. Therefore, we should increase the recovery speed of the pessimistic. If we can control the recovery speed of the pessimistic, we can effectively reduce the number of pessimistic subjects and then effectively control and predominate the emergency trend to reduce the probability of emergencies.

**Figure 6:**
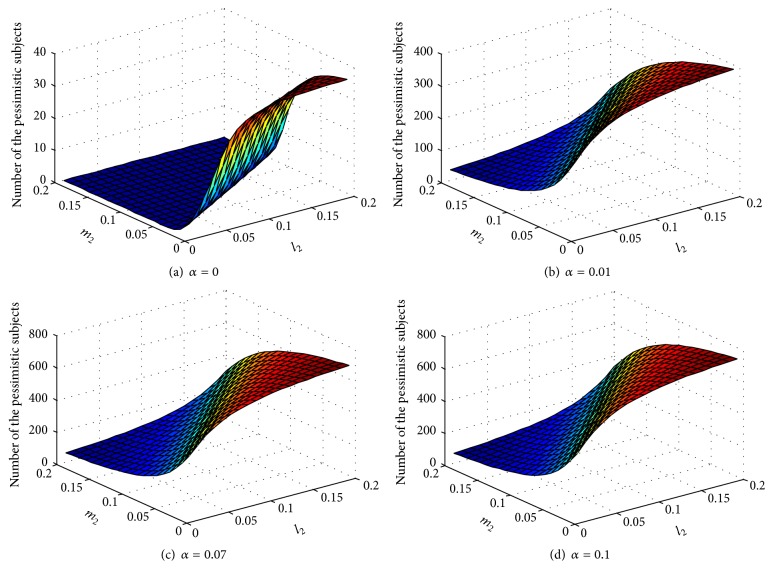
Effect of spontaneous generation speed on equilibrium.

We assume that the possibility of the optimistic coming in contact with the pessimistic subjects is different from that of the pessimistic subjects coming in contact with the optimistic subjects. We then determine how these two possibilities affect the equilibrium number of the pessimistic subjects under different parameters.  Figure 6(a) shows that a fast speed of spontaneous generation results in a great number of the pessimistic subjects. The number of the pessimistic subjects in equilibrium is positively related to the possibility that the optimistic subjects will be infected as pessimistic subjects (*l*_2_) but is negatively related to the possibility that the pessimistic subjects will be infected as optimistic subjects (*m*_2_). As shown in Figure 6(b), when the value of parameter *α* is small, an insignificant difference in the infection rate (*m*_2_ and *l*_2_) may result in a tremendous difference in the number of the pessimistic subjects in equilibrium. However, when the spontaneous generation reaches a certain extent, the changes in the number of the pessimistic subjects in equilibrium are not obvious. By comparing Figures 6(c) and 6(d), we can distinguish an inconsiderable difference in the pessimistic in equilibrium under the parameters *α* = 0.07 and *α* = 0.1.

Figure 6 illustrates that the number of parameters increases significantly with the increasing value of *α*. The spontaneous generation speed can clearly affect the emotional contagion. In other words, if we do not control the spontaneous generation speed, more and more people will become pessimistic, while the group emotion of pessimistic subjects is important in reality. Therefore, we must pay attention to the range of spontaneous generation speed for any emergency issues.

## 4. Discussion

This study adds a new relationship between the pessimistic subjects and the optimistic subjects based on the SISa model and modifies the SOSa-SPSa model, which aims to discover the effect of interaction on the number of the pessimistic subjects in equilibrium and to evaluate the regulating effects of different parameters. The simulation results of the modified SOSa-SPSa model can reach the equilibrium state using the parameters obtained from the experimental data of Hill et al. (2010). The simulation results of different parameters present the following new discoveries: (1) The recovery speed of the pessimistic subjects has an obvious regulative effect on the number of the pessimistic subjects in the final equilibrium. (2) The possibility of the optimistic coming in contact with the pessimistic subjects to become pessimistic plays a greater role than that of reverting to the susceptible in the number of the pessimistic subjects in equilibrium. (3) The number of the pessimistic subjects in equilibrium is positively related to the possibility that the optimistic subjects coming in contact with the pessimistic subjects will be infected as pessimistic subjects but is negatively related to the possibility that the pessimistic subjects coming in contact with the optimistic subjects will be infected as optimistic subjects. (4) When the speed of spontaneous generation is slow, the number of the pessimistic subjects in equilibrium sharply increases. However, the increase is not apparent when the speed of spontaneous generation reaches a certain number.

The modified model proposed in this study can vividly depict the entire process of sentiment contagion involving two opposite states, namely, the pessimistic subjects and the optimistic subjects. The simulation results are also applicable to actual reality. Nevertheless, a limitation of this study is the lack of analysis on the stability of the equilibrium of the modified SOSa-SPSa model. The model has 12 parameters. Consequently, analyzing the equilibrium stability is difficult. However, the discussion is indeed inevitable. Another drawback of this study is the uncertainty in the state to which the contacts (the pessimistic subjects or the optimistic subjects) will revert when confronting the people with opposite sentiments (the optimistic subjects or the pessimistic subjects). This uncertainty requires further study.

The new model can also be used to study and supervise the emotion of a crowd, especially when a large-scale emergency occurs. The model is built on the basis of the simplest situation; a complicated atmosphere and interpersonal relationships are not considered. Therefore, if we want to apply this model to reality, we should determine the effect of special individuals, such as the leaders of a crowd or the rescuers of a disaster [20, 21]. In this study, we assume that individuals are not different from one another and that they are deindividuations. We ignore their personality, which may be a crucial factor to feel the emotion of others or to express to others [22]. Furthermore, we should pay attention to the influence of the media because rumors and social unrest can easily break out if the media is used improperly.
